# The Role of Comorbid Diseases in Geographic Disparities of Mortality From Septicemia Among Older Adults Across the United States

**DOI:** 10.1093/geroni/igaa057.460

**Published:** 2020-12-16

**Authors:** Julia Kravchenko, Igor Akushevich

**Affiliations:** Duke University, Durham, North Carolina, United States

## Abstract

Sepsis is a prominent cause of death in the US. However, few studies examined its regional variations. Using the Multiple-Cause-of-Death and 5%-Medicare data, we analyzed mortality among the US patients aged 65+ who had septicemia and various comorbid diseases in the states with the highest (HI,FL,AZ,CT,CO, and MN––a.k.a. leading states) and lowest (AR,TN, LA,OK,KY,AL,MS, and WV–a.k.a. lagging states) life expectancy. In 2010-2018, age-adjusted mortality from septicemia was almost twice as high in lagging (85.2±0.5/100,000) comparing to leading (45.2±0.3/100,000) states. Between-the-states difference was the most pronounced at age 65-74 (44.7±0.4 vs. 20.8±0.2) and gradually reduced in older age groups. Higher mortality from septicemia in the lagging US states could be, at least in part, because of poorer health of the beneficiaries who enter Medicare at age 65. Mortality rates were the highest among septicemia patients who had comorbid cancer, ischemic heart disease, influenza/pneumonia, or kidney failure. Mortality rates of patients with co-existing Alzheimer’s disease or arterial hypertension were lower, but these comorbidities contributed most to geographic disparities. The role of complications due to surgical and other medical procedures in disparities across the states was minimal. Substantial contributions to geographic disparities in septicemia mortality were detected not only for well-known and highly-lethal comorbidities such as diabetes, heart failure, and kidney diseases, but also for less frequently discussed conditions with lower per se mortality such as disease of intestine, skin/subcutaneous tissue, and cystitis, urethritis, or unspecified infections of urinary tract.

